# The Relationship of Large-Artery Atherothrombotic Stroke with Plasma Trimethylamine N-Oxide Level and Blood Lipid-Related Indices: A Cross-Sectional Comparative Study

**DOI:** 10.1155/2021/5549796

**Published:** 2021-04-22

**Authors:** Dongjuan Xu, Wenfeng Zhao, Juexian Song, Lu Yin, Kun Wang, Lianyan Wei, Yunyun Xu, Hongfei Li, Baoquan Min, Ning Tang, Xiaoyan Jiang, Hui Liu, Shuo Yan, Haixia Leng, Qing Xue, Mao Peng, Hongxing Wang

**Affiliations:** ^1^Department of Neurology, Dongyang People's Hospital, Wenzhou Medical University, Zhejiang, China 322100; ^2^Department of Neurology, Xuanwu Hospital, Capital Medical University, Beijing, China 100053; ^3^Medical Research & Biometrics Centre, National Centre for Cardiovascular Diseases, Fuwai Hospital, Peking Union Medical College & Chinese Academy of Medical Sciences, Beijing, China 102300; ^4^Department of Neurology, Beijing Puren Hospital, Beijing, China 100062; ^5^Beijing Psychosomatic Disease Consultation Center, Xuanwu Hospital, Capital Medical University, Beijing, China 100053; ^6^Department of Neurology, The First People's Hospital of Yunnan Province, The Affiliated Hospital of Kunming University of Science and Technology, Yunnan, China 650032; ^7^Department of Neurology, Shexian Hospital of Hebei Province, Hebei, China 056400; ^8^Department of Neurology, Rongcheng Hospital of Hebei Province, Hebei, China 071700; ^9^Department of Neurology, The Third Affiliated Hospital of Shenzhen University, Guangdong, China 518000; ^10^Institute of Sleep and Consciousness Disorders, Beijing Institute for Brain Disorders, Capital Medical University, Beijing, China 100053

## Abstract

**Objective:**

The role of trimethylamine N-oxide (TMAO) in cardiovascular diseases has been highlighted. Nevertheless, the associations of large-artery atherosclerotic (LAA) stroke with TMAO and blood lipid-related indices are little investigated.

**Methods:**

A cross-sectional comparative study was performed on 50 patients with LAA stroke and 50 healthy controls. Basic demographic data, common vascular risk factors, and blood lipid-related indices were collected. Plasma TMAO was detected through liquid chromatography tandem mass spectrometry. Multivariable unconditional logistic regression analyses were run to assess the associations of LAA stroke with plasma TMAO level and blood lipid-related indices. The area under the curve (AUC) of the receiver operating characteristic (ROC) was computed to assess the diagnostic performance of plasma TMAO level and blood lipid-related indices for LAA stroke.

**Results:**

Compared with healthy controls, the elevated plasma TMAO level (odds ratio [OR], 7.03; 95% confidence interval [CI], 2.86, 17.25; *p* < 0.01) and Apo-B (OR, 1.74; 95% CI, 1.06, 2.85; *p* = 0.03) were observed in LAA stroke patients, while lower Apo-A1 (OR, 0.56; 95% CI, 0.34, 0.91; *p* = 0.02), Apo-A1 to Apo-B ratio (OR, 0.29; 95% CI, 0.15, 0.56; *p* < 0.01), and HDL-C (OR, 0.56; 95% CI, 0.35, 0.91; *p* = 0.02) were found in LAA stroke patients after adjusted for age and gender. Moreover, plasma TMAO (AUC, 0.89; 95% CI, 0.83, 0.95), Apo-A1 (AUC, 0.81; 95% CI, 0.72, 0.89), Apo-B (AUC, 0.81; 95% CI, 0.73, 0.90), Apo-A1 to Apo-B ratio (AUC, 0.85; 95% CI, 0.78, 0.93), and HDL-C (AUC, 0.81; 95% CI, 0.72, 0.89) showed good diagnostic values for LAA stroke in adjusted models.

**Conclusions:**

The plasma TMAO level, Apo-A1, Apo-B, and HDL-C are important biomarkers for LAA stroke patients.

## 1. Introduction

Stroke is a significant cause of death worldwide [1], and large-artery atherosclerosis is a significant cause of ischemic stroke [2, 3], approximately 15% of all ischemic strokes [2]. Large-artery atherosclerotic (LAA) stroke is one common stroke in China [3], and the most common areas orderly are the basilar artery, the internal carotid arteries, the middle cerebral arteries, the intracranial vertebral arteries, the posterior cerebral arteries, and the anterior cerebral arteries [3]. Atherosclerosis is a pathologic process causing LAA stroke and the most common cause of in situ regional diseases within the extracranial and intracranial arteries that provide the brain [3]. Many factors are involved in the pathogenesis of atherosclerosis [3, 4]. Of them, lipid abnormality plays a critical role in the development of atherosclerosis [4, 5].

Recently, more attention on the association of plasma trimethylamine *N*-oxide (TMAO) with the pathogenesis of cardiovascular disease has been attracted [6–10]. Previous reports showed that plasma TMAO, produced in the liver, has associations with cardiovascular disease [11], atherothrombotic diseases [7], ischemic brain injury secondary to carotid artery stenting [12], and stroke severity and infarct volume in patients with acute ischemia [13]. Plasma TMAO directly causes platelet hyperreactivity and increases thrombosis risks in animal models and healthy subjects [14]. It may also indicate coronary plaque vulnerability and development in patients with coronary artery disease [15]. Moreover, it is associated with atherosclerosis formation [10, 16–18], which causes the stenosis of large artery as the reason for LAA stroke.

On the above grounds, we speculate that plasma TMAO level and blood lipid-related indices may be associated with LAA stroke patients. Therefore, in this cross-sectional comparative study, we aimed to investigate whether circulating TMAO level and blood lipid-related indices are associated with LAA stroke.

## 2. Methods

### 2.1. Study Design and Participants

This comparative study was performed from October 2018 to November 2019. All subjects were enrolled from two research centers in China (Dongyang People's Hospital of Wenzhou Medical University and Xuanwu Hospital of Capital Medical University). The local Ethics Committees of Xuanwu Hospital, Capital Medical University (LYS2018008), and Dongyang People's Hospital, Wenzhou Medical University (2017-KY-036), approved the study protocols. All subjects and/or their legal representatives provided written informed consent.

The 50 LAA stroke patients who suffered acute cerebral ischemia, meeting the stroke diagnostic criteria formulated by the Chinese cerebrovascular disease classification and were validated by magnetic resonance imaging (MRI) and/or computed tomography (CT) scan [19], were sequentially enrolled. The inclusion criteria were as follows: (1) aged 18 years or older, (2) Han Chinese, (3) no limitation on gender, (4) LAA stroke was diagnosed based on the TOAST classification with evidence of cerebral infarction confirmed by MRI and/or CT [20], and (5) carotid artery stenosis with a narrowing greater than 70% lumen were responsible for these LAA stroke patients [21]. The exclusion criteria included the following: (1) other types of cerebrovascular diseases (e.g., cerebral hemorrhage, transient ischemic attack, cerebral aneurysm, or cerebrovascular malformation), (2) severe systemic diseases (such as a tumor and infectious or inflammatory diseases), (3) known embolic source (aortic arch, cardiac, or carotid), (4) consumption with probiotics or antibiotics within one month before admission, contraindications to MRI (e.g., any implanted metal devices), (5) participation in any clinical study concurrently, (6) inability to understand the study, (7) severe physical diseases (including congestive cardiac failure, respiratory failure, renal failure, and liver dysfunction). The enrolled patients received the best medical management in the stroke centers. All patients received acute treatment and secondary prevention of stroke according to Chinese guidelines during hospitalization and after discharge.

50 healthy controls were recruited from relatives of outpatients or inpatients (including their immediate family members). Their inclusion criteria were as follows: (1) aged 18 years or older, (2) Han Chinese, (3) no limitation on gender, (4) no history of cerebrovascular diseases, (5) no history of atherosclerotic diseases in the carotid arteries, (6) no the atrial fibrillation, and (7) regular physical examinations. Exclusion criteria were not different from the exclusion criteria of the stroke patient group.

### 2.2. Measurement of Blood Lipid-Related Indices and Plasma TMAO Level

The overnight fasting blood samples were collected for routine blood lipid-related indices at 6: 00 to 7: 00 AM before breakfast. Plasma was prepared from the blood of EDTA tube and kept at -80°C until examination. Plasma TMAO level was qualified via stable isotope dilution liquid chromatography tandem mass spectrometry, consistent with the previous reported [12, 13]. In brief, the mixture of 80 *μ*L, 10 *μ*mol/L d9-TMAO, and 20 *μ*L plasma was vortexed for 1 minute. The supernatant was centrifuged at 15,000 × g at 4°C for 25 min and then transferred to a new sample bottle for the check. The 10 mL of supernatant was injected directly into a silica column at a flow rate of 0.8 mL/min with 80% A (0.1% formic acid in water) and 20% B (methanol). TMAO and d9-TMAO were detected by the positive multiple reaction monitoring mass spectrometry mode by characteristic precursor–production transitions including m/z 76/58 and m/z 85/66. For detection of TMAO concentration, a standard curve using multiknown concentrations of TMAO was used. The median (interquartile range) of healthy participants' reference values was 2.8 (1.9-4.8) *μ*mol/L. The intra-assay and interassay coefficients of variation were 1.9%-5.6% and 2.9%-8.4%, respectively. Testers were blind to all subjects' clinical data.

### 2.3. Calculation of the Volume of Infarct and the Area of Carotid Atherosclerotic Plaque

Imaging acquisition details were standardized in both sites. The area of carotid artery plaque was measured via the common color Doppler ultrasound system by measuring the areas of unstable and stable carotid artery plaques. All patients received MRI within 24 hours of hospitalization with a 1.5 T or 3.0 T scanner. The sequence includes axial spin-echo T1-weighted sequence, T2-weighted sequence, fluid-attenuated inversion recovery sequence, DWI sequence (*b* = 1,000 s/mm^2^, 2 mm isotropic resolution, and 30 diffusion directions), and 3D time-of-flight angiography. The slice layer of MRI was 5 mm, and the slice interval was 1.5 mm. Acute cerebral infarction was defined as high signal intensity on the DWI sequence. Infarct areas were manually qualified on DWI sequences. The images were collected and analyzed by experienced stroke neurologists who were blind to the participants' other characteristics. Infarct volumes were computed by slice layer multiplied by infarct area in each slice.

### 2.4. Statistical Analysis

Statistical tests were conducted using SAS, version 9.4 (SAS Institute Inc). Chi-square test or Fisher's exact test was used to compare group differences of categorical variables. Mann-Whitney *U* test was used to compare continuous variables. Multivariate unconditional logistic regressions were performed to evaluate the associations of LAA stroke with plasma TMAO and blood lipid-related indices (i.e., lipoprotein-a (Lp-a), apolipoprotein A1 (Apo-A1), apolipoprotein B (Apo-B), Apo-A1/B ratio, total cholesterol (TC), high-density lipoprotein cholesterol (HDL-C), low-density lipoprotein cholesterol (LDL-C), triglycerides (TG), D-Dime, and thrombin time (TT)). Model 1 was unadjusted. Model 2 was adjusted for sex and age. The area under the curves (AUCs) of the receiver operating characteristic (ROC) were also calculated to assess their diagnostic performance of these abovementioned indices. Correlations between plasma TMAO and Lp-a, Apo-A1, Apo-B, Apo-A1/B ratio, TC, HDL-C, LDL-C, TG, D-Dimer, and TT were calculated using Spearman's correlation.

## 3. Results

### 3.1. General Characteristics

50 LAA stroke patients and 50 healthy controls were included in the study.  Table 1 demonstrated their demographics, vascular risk factors, plasma TMAO, and blood lipid-related indices in stroke patients and healthy controls. LAA stroke patients showed a higher TMAO than that of healthy controls (LAA vs. control: 2659.51 ± 976.81 ng/mL vs. 1484.75 ± 648.87 ng/mL, *p* < 0.01). Significance was also observed between LAA stroke patients and healthy controls in age, gender, smoking, alcohol, diabetes, and hypertension, the volume of acute cerebral infarction, Lp-a, Apo-A1, Apo-B, the ratio of Apo-A1/B, HDL-C, and D-Dimer (*p* < 0.05), while no statistical differences were found in obesity, area of carotid artery plaque, TC, LDL-C, TG, and TT (*p* > 0.05).

### 3.2. The Associations of the Risk of LAA Stroke with Plasma TMAO and Blood Lipid-Related Indices (Table 2)

The multivariate unconditional logistic regression analyses revealed that elevated plasma TMAO was associated with higher risk of LAA stroke (odds ratio [OR], 3.99; 95% confidence interval [CI], 2.16, 7.36; *p* < 0.01), while lower Apo-A1 (OR, 0.53; 95% CI, 0.34, 0.83; *p* = 0.01), lower Apo-A1 to Apo-B ratio (OR, 0.30; 95% CI, 0.17, 0.53; *p* < 0.01), lower TC (OR, 0.72; 95% CI, 0.54, 0.96; *p* < 0.01), and lower HDL-C (OR, 0.61; 95% CI, 0.40, 0.93; *p* < 0.01) were associated with higher risks of LAA stroke patients in an unadjusted model.

After adjusting for sex and age, logistic regression exhibited that higher plasma TMAO level (OR, 7.03; 95% CI, 2.86, 17.25; *p* < 0.01) and higher Apo-B (OR, 1.74; 95% CI, 1.06, 2.85; *p* = 0.03) were associated with higher risks of LAA stroke, while lower Apo-A1 (OR, 0.56; 95% CI, 0.34, 0.91; *p* = 0.02), lower Apo-A1 to Apo-B ratio (OR, 0.29; 95% CI, 0.15, 0.56; *p* < 0.01), and lower HDL-C (OR, 0.56; 95% CI, 0.35, 0.91; *p* = 0.02) were observed in LAA stroke patients.

### 3.3. The Diagnostic Performance of Plasma TMAO and Blood Lipid-Related Indices for LAA Stroke (Table 2)

The AUCs were calculated to assess the diagnostic ability of plasma TMAO and other blood lipid-related indices for vulnerable LAA stroke. The elevated plasma TMAO (AUC, 0.79; 95% CI, 0.70, 0.88) and lower Apo-A1 to Apo-B ratio (AUC, 0.75; 95% CI, 0.66, 0.85) were found to have good diagnostic performance for vulnerable LAA stroke in an unadjusted model.

While adjusted for sex and age in multivariate unconditional logistic regression, the elevated plasma TMAO (AUC, 0.89; 95% CI, 0.83, 0.95) and the increased Apo-B (AUC, 0.81; 95% CI, 0.73, 0.90) were of significant diagnostic values for vulnerable LAA stroke. By contrast, the lower Apo-A1 (AUC, 0.81; 95% CI, 0.72, 0.89), the lower Apo-A1 to Apo-B ratio (AUC, 0.85; 95% CI, 0.78, 0.93), and lower HDL-C (AUC, 0.81; 95% CI, 0.72, 0.89) showed good diagnostic ability for suffering LAA stroke.

### 3.4. Correlation Analysis between Plasma TMAO Level and Blood Lipid-Related Indices in LAA Stroke Patients and Healthy Controls

**Figure 1:**
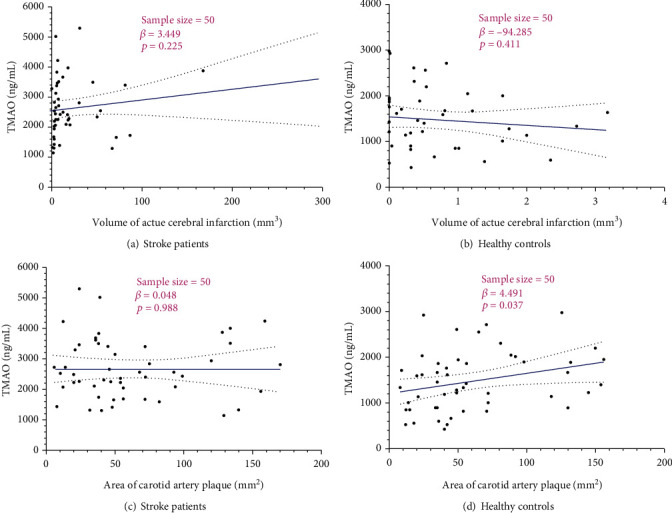
Scatter plots for plasma TMAO with volume of acute cerebral infarction and area of carotid artery plaque in LAA stroke patients and healthy controls by Spearman's correlation.

Spearman's correlation analysis revealed that plasma TMAO was not significantly correlated with volume of acute cerebral infarction and area of carotid artery plaque in LAA stroke patients, but positively correlated with area of carotid artery plaque in healthy controls (*β* = 4.49, *p* = 0.04) (Figure 1). Furthermore, there was no relationship between plasma TMAO and various blood lipid-related indices, including Lp-a, Apo-A1, Apo-B, the ratio of Apo-A1/B, TC, HDL-C, LDL-C, and TC (*p* > 0.05) (Table 3).

## 4. Discussion

The main findings of this study include plasma TMAO level in LAA stroke patients was significantly higher than that of the control group. Furthermore, for LAA stroke patients, the increased plasma TMAO level and Apo-B were risk factors, while lower Apo-A1, Apo-A1 to Apo-B ratio, and HDL-C were risk factors. Moreover, the increased plasma TMAO level and Apo-B were good diagnostic values for susceptible LAA stroke, but lower Apo-A1, Apo-A1 to Apo-B ratio, and HDL-C were good diagnostic values for suffering LAA stroke. Interestingly, the elevated plasma TMAO level was positively correlated with the area of carotid artery plaque in healthy controls, but not associated with the volume of acute cerebral infarction and area of carotid artery plaque in LAA stroke patients.

Our results also replicated the previous reports on the risk factors included age, smoking, alcohol drinking, diabetes, and hypertension [3, 22] and blood lipid abnormalities in LAA stroke patients [5, 10]. Most importantly, compared with healthy controls, LAA stroke patients had higher plasma TMAO levels, which indicated that TMAO might play a critical role in the pathophysiological mechanism of patients with stroke having large-artery atherosclerosis, and supported the need for further study of TMAO in the pathophysiological mechanism related to LAA stroke. This result aligned with the previous report [13] and the statement that the increased TMAO may promote platelet hyperreactivity and increase thrombosis risk [14].

In our study, the result that the elevated plasma TMAO and Apo-B were found to be risk factors for LAA stroke may be important manageable biological markers for exploring the potential of having LAA stroke in those with large-artery atherosclerosis. These markers combined with other factors on blood lipid-related indices, such as Apo-A1, Apo-A1 to Apo-B ratio, and HDL-C, may reflect the progress of pathogenesis of LAA stroke and be a panel of assessments for individuals with large-artery atherosclerosis without stroke. It has been reported that plasma TMAO is a potential mediator of the pathogenesis of atherosclerotic diseases, including stoke and myocardial infarction [23]. Our findings further revealed that the elevated plasma TMAO and blood indictors reflecting the atherosclerosis such as Apo-A1, Apo-B, and HDL-C had good diagnostic performance for those vulnerably suffered LAA stroke, which added to the existing evidence presenting that TMAO level may regard as a useful and potential prognostic biological indictor in LAA stroke, even beyond the currently clinic routine check.

Our study did not reveal that the elevated plasma TMAO was positively correlated with the area of carotid artery plaque in healthy controls but not significantly correlated with volume of acute cerebral infarction and area of carotid artery plaque in LAA stroke patients. The previous study supported this point of our results [24]. It showed no direct association between the increased plasma TMAO and the extent of atherosclerosis in mice and humans. Still, there was an association of the increased plasma TMAO levels with atherosclerotic plaque instability in a mouse model of plaque instability [24]. In contrast, other studies found that plasma TMAO has relations with atherosclerosis [25–27]. Interestingly, in our study, the elevated plasma TMAO levels were not associated with all blood lipid-related indices in our study in LAA stoke patients, suggesting the associations of plasma TMAO and blood lipids still need to be further noted and investigated for stroke.

The strengths of the study include a homogeneous LAA stroke population, blind evaluation on TMAO and imaging collection and analysis, and simultaneous detection of multiple blood lipid indicators. The study has limitations. Firstly, the sample was not big in both groups; the results of the current study should be confirmed in a large sample. Secondly, the positive control group, such as different stroke types, was a lack in this study. The positive control group of stroke, except for LAA stroke, should be designed for exploring the role of plasma TMAO and the associations of TMAO and blood lipid-related indices in various types of stroke, including different LAA strokes with various responsible large arteries. Lastly, the potential factors on plasma TMAO level, including diet, gut microbiota, drug management, and liver flavin monooxygenase [6, 7, 24, 28, 29], should be considered in different strokes of future studies.

## 5. Conclusions

To be our best knowledge, we firstly explored the association of LAA stroke with plasma TMAO and blood lipid-related indices. We demonstrated that plasma TMAO level, Apo-A1, Apo-B, and HDL-C could be as the potential biomarkers on LAA stroke and in blood will be beneficial for early managing and preventing stroke [30], especially LAA stroke.

## Figures and Tables

**Table 1:** General characteristics.

Characteristics	Stroke patients (*n* = 50)	Healthy controls (*n* = 50)	*p* ^1^
Male sex, % (*n*)	70.00 (35)	28.00 (14)	<0.01
Age, years, mean ± SD	63.20 ± 12.00	57.36 ± 10.65	0.01
Overweight or obesity, % (*n*)	32.00 (16)	20.00 (10)	0.43
Smoking, % (*n*)	38.00 (19)	4.00 (2)	<0.01
Alcohol, % (*n*)	32.00 (16)	10.00 (5)	<0.01
Diabetes, % (*n*)	42.00 (21)	8.00 (4)	<0.01
Hypertension, % (n)	72.00 (36)	26.00 (13)	<0.01
Volume of acute cerebral infarction, mm^3^, mean ± SD	25.02 ± 49.50	0.68 ± 0.82	<0.01
Area of carotid artery plaque, mm^2^, mean ± SD	61.42 ± 44.27	60.67 ± 42.71	0.95
TMAO, ng/mL, mean ± SD	2659.51 ± 976.81	1484.75 ± 648.87	<0.01
Lp-a, g/L, mean ± SD	131.52 ± 90.83	33.38 ± 10.50	<0.01
Apo-A1, g/L, mean ± SD	1.04 ± 0.22	1.18 ± 0.26	<0.01
Apo-B, g/L, mean ± SD	1.00 ± 0.26	0.83 ± 0.28	<0.01
Apo-A1/B ratio, mean ± SD	1.03 ± 0.27	1.43 ± 0.42	<0.01
TC, mmol/L, mean ± SD	4.70 ± 1.12	4.37 ± 0.81	0.12
HDL-C, mmol/L, mean ± SD	1.02 ± 0.25	1.16 ± 0.28	<0.01
LDL-C, mmol/L, mean ± SD	2.91 ± 1.02	2.57 ± 0.80	0.07
TG, mmol/L, mean ± SD	1.78 ± 1.08	1.49 ± 0.76	0.16
D-Dimer, IU/L, mean ± SD	0.88 ± 0.84	0.48 ± 0.33	<0.01
TT, s, mean ± SD	16.01 ± 0.88	16.15 ± 0.89	0.37

Abbreviations: SD: standard deviation; TMAO: trimethylamine N-oxide; Lp-a: lipoprotein-a; Apo-A1: apolipoprotein A1; Apo-B: apolipoprotein B; TC: total cholesterol; HDL-C: high-density lipoprotein cholesterol; LDL-C: low-density lipoprotein cholesterol; TG: triglycerides; TT: thrombin time. ^1^*p* value was obtained using chi-square tests or Fisher's exact tests for categorical variables and Mann-Whitney *U* tests for continuous variables.

**Table 2:** Odds ratios and areas under the curve for stroke risk per one sex-specific standard deviation increase of parameters.

Parameters	Unadjusted model			Adjusted model^1^		
	OR (95% CI)	*p* value	AUC (95% CI)	OR (95% CI)	*p* value	AUC (95% CI)
TMAO (ng/mL)	3.99 (2.16, 7.36)	<0.01	0.79 (0.70, 0.88)	7.03 (2.86, 17.25)	<0.01	0.89 (0.83, 0.95)
Area of carotid artery plaque (mm^2^)	1.07 (0.72, 1.59)	0.74	0.52 (0.40, 0.63)	1.00 (0.63, 1.59)	0.99	0.78 (0.69, 0.88)
Apo-A1 (g/L)	0.53 (0.34, 0.83)	0.01	0.67 (0.57, 0.78)	0.56 (0.34, 0.91)	0.02	0.81 (0.72, 0.89)
Apo-B (g/L)	1.43 (0.95, 2.17)	0.09	0.61 (0.50, 0.72)	1.74 (1.06, 2.85)	0.03	0.81 (0.73, 0.90)
Apo-A1 to Apo-B ratio	0.30 (0.17, 0.53)	<0.01	0.75 (0.66, 0.85)	0.29 (0.15, 0.56)	<0.01	0.85 (0.78, 0.93)
TC (mmol/L)	0.72 (0.54, 0.96)	0.02	0.65 (0.54, 0.76)	1.32 (0.83, 2.10)	0.24	0.79 (0.70, 0.88)
HDL-C (mmol/L)	0.61 (0.40, 0.93)	0.02	0.64 (0.54, 0.75)	0.56 (0.35, 0.91)	0.02	0.81 (0.72, 0.89)
LDL-C (mmol/L)	0.80 (0.56, 1.15)	0.23	0.59 (0.48, 0.70)	1.21 (0.77, 1.89)	0.41	0.78 (0.69, 0.88)
TG (mmol/L)	0.89 (0.62, 1.28)	0.52	0.55 (0.43, 0.66)	1.50 (0.91, 2.47)	0.11	0.80 (0.71, 0.89)

Abbreviations: OR: odds ratio; CI: confidence interval; AUC: area under the curve; TC: total cholesterol; HDL-C: high-density lipoprotein cholesterol; LDL-C: low-density lipoprotein cholesterol; TG: triglycerides. ^1^Adjusted for age and gender.

**Table 3:** Correlations between plasma TMAO and blood lipid-related indices in stroke patients and healthy participants.

Parameters	Stroke patients		Healthy participants	
	Beta (*β*)	*p* value	Beta (*β*)	*p* value
Lp-a (g/L)	1.44	0.36	-0.82	0.93
Apo-A1 (g/L)	-59.05	0.93	-513.08	0.16
Apo-B (g/L)	-255.90	0.64	-391.99	0.25
Apo-A1 to Apo-B ratio	578.09	0.27	-163.48	0.47
TC (mmol/L)	-57.75	0.65	-32.02	0.78
HDL-C (mmol/L)	335.33	0.55	-521.03	0.12
LDL-C (mmol/L)	-32.36	0.82	-132.46	0.26
TG (mmol/L)	-178.22	0.17	-81.93	0.51

Abbreviations: TMAO: trimethylamine N-oxide; Apo-A1: apolipoprotein A1; Apo-B: apolipoprotein B; TC: total cholesterol; HDL-C: high-density lipoprotein cholesterol; LDL-C: low-density lipoprotein cholesterol; TG: triglycerides.

## Data Availability

The data in this manuscript is available from the first or corresponding author on reasonable request.

## References

[1] Xu D., Chu X., Wang K. (2021). Potential factors for psychological symptoms at three months in patients with young ischemic stroke. *Bio Med Research International*.

[2] Cole J. W. (2017). Large artery atherosclerotic occlusive disease. *Continuum (Minneapolis, Minn)*.

[3] Qureshi A. I., Caplan L. R. (2014). Intracranial atherosclerosis. *Lancet*.

[4] Rahman M. S., Woollard K. (2017). Atherosclerosis. *Advances in Experimental Medicine and Biology*.

[5] Steinberg D., Witztum J. L. (2010). Oxidized low-density lipoprotein and atherosclerosis. *Arteriosclerosis, Thrombosis, and Vascular Biology*.

[6] Wang Z., Klipfell E., Bennett B. J. (2011). Gut flora metabolism of phosphatidylcholine promotes cardiovascular disease. *Nature*.

[7] Koeth R. A., Wang Z., Levison B. S. (2013). Intestinal microbiota metabolism of L-carnitine, a nutrient in red meat, promotes atherosclerosis. *Nature Medicine*.

[8] Wu W. K., Chen C. C., Liu P. Y. (2019). Identification of TMAO-producer phenotype and host-diet-gut dysbiosis by carnitine challenge test in human and germ-free mice. *Gut*.

[9] Wang X., Li X., Dong Y. (2020). Vitamin D decreases plasma trimethylamine-N-oxide level in mice by regulating gut microbiota. *BioMed Research International*.

[10] Canyelles M., Tondo M., Cedó L., Farràs M., Escolà-Gil J., Blanco-Vaca F. (2018). Trimethylamine N-oxide: a link among diet, gut microbiota, gene regulation of liver and intestine cholesterol homeostasis and HDL function. *International Journal of Molecular Sciences*.

[11] Tang W. H., Wang Z., Levison B. S. (2013). Intestinal microbial metabolism of phosphatidylcholine and cardiovascular risk. *The New England Journal of Medicine*.

[12] Wu C., Li C., Zhao W. (2018). Elevated trimethylamine N-oxide related to ischemic brain lesions after carotid artery stenting. *Neurology*.

[13] Wu C., Xue F., Lian Y. (2020). Relationship between elevated plasma trimethylamine N-oxide levels and increased stroke injury. *Neurology*.

[14] Zhu W., Gregory J. C., Org E. (2016). Gut microbial metabolite TMAO enhances platelet hyperreactivity and thrombosis risk. *Cell*.

[15] Fu Q., Zhao M., Wang D. (2016). Coronary plaque characterization assessed by optical coherence tomography and plasma trimethylamine-N-oxide levels in patients with coronary artery disease. *The American Journal of Cardiology*.

[16] Warrier M., Shih D. M., Burrows A. C. (2015). The TMAO-generating enzyme flavin monooxygenase 3 is a central regulator of cholesterol balance. *Cell Reports*.

[17] Ding L., Chang M., Guo Y. (2018). Trimethylamine-N-oxide (TMAO)-induced atherosclerosis is associated with bile acid metabolism. *Lipids in Health and Disease*.

[18] Bennett B. J., Vallim T. Q. A., Wang Z. (2013). Trimethylamine-N-oxide, a metabolite associated with atherosclerosis, exhibits complex genetic and dietary regulation. *Cell Metabolism*.

[19] Chinese Society of Neurology (2017). Chinese cerebrovascular disease classification 2015. *Chinese Journal of Neurology*.

[20] Adams H. P., Bendixen B. H., Kappelle L. J. (1993). Classification of subtype of acute ischemic stroke. Definitions for use in a multicenter clinical trial. TOAST. Trial of Org 10172 in Acute Stroke Treatment. *Stroke*.

[21] Barnett H. J. M., Taylor D. W., Haynes R. B., North American Symptomatic Carotid Endarterectomy Trial Collaborators (1991). Beneficial effect of carotid endarterectomy in symptomatic patients with high-grade carotid stenosis. *The New England Journal of Medicine*.

[22] Ritz K., Denswil N. P., Stam O. C., van Lieshout J. J., Daemen M. J. A. P. (2014). Cause and mechanisms of intracranial atherosclerosis. *Circulation*.

[23] Zhai Q., Wang X., Chen C. (2019). Prognostic value of plasma trimethylamine N-oxide levels in patients with acute ischemic stroke. *Cellular and Molecular Neurobiology*.

[24] Koay Y. C., Chen Y. C., Wali J. A. (2021). Plasma levels of trimethylamine-N-oxide can be increased with 'healthy' and 'unhealthy' diets and do not correlate with the extent of atherosclerosis but with plaque instability. *Cardiovascular Research*.

[25] Yamashita T. (2017). Intestinal immunity and gut microbiota in atherogenesis. *Journal of Atherosclerosis and Thrombosis*.

[26] Wilson A., McLean C., Kim R. B. (2016). Trimethylamine-N-oxide: a link between the gut microbiome, bile acid metabolism, and atherosclerosis. *Current Opinion in Lipidology*.

[27] Duttaroy A. K. (2021). Role of gut microbiota and their metabolites on atherosclerosis, hypertension and human blood platelet function: a review. *Nutrients*.

[28] Janeiro M. H., Ramírez M. J., Milagro F. I., Martínez J., Solas M. (2018). Implication of trimethylamine N-oxide (TMAO) in disease: potential biomarker or new therapeutic target. *Nutrients*.

[29] Fu B. C., Hullar M. A. J., Randolph T. W. (2020). Associations of plasma trimethylamine N-oxide, choline, carnitine, and betaine with inflammatory and cardiometabolic risk biomarkers and the fecal microbiome in the multiethnic cohort adiposity phenotype study. *The American Journal of Clinical Nutrition*.

[30] Bustamante A., López-Cancio E., Pich S. (2017). Blood biomarkers for the early diagnosis of stroke: the stroke-chip study. *Stroke*.

